# Effect of canola oil supplementation level on total tract digestion, ruminal fermentation, and methane emissions of cows grazing *Urochloa* sp. supplemented with a fixed amount of concentrate

**DOI:** 10.1007/s11250-023-03485-8

**Published:** 2023-02-11

**Authors:** Jonathan Noe Avilés-Nieto, Claudia Cecilia Márquez-Mota, Juan Hebert Hernández-Medrano, Jacinto Efrén Ramírez-Bribiesca, Epigmenio Castillo-Gallegos, Alejandro Plascencia, Francisco Alejandro Castrejón-Pineda, Luis Corona

**Affiliations:** 1 Facultad de Medicina Veterinaria y Zootecnia, Departamento de Nutrición Animal y Bioquímica, Universidad Nacional Autónoma de México, Ciudad de México, México; 2 Faculty of Veterinary Medicine, University of Calgary, Calgary, AB Canada; 3 Departamento de Ganadería, Colegio de Postgraduados, Campus Montecillo, Estado de México, México; 4 Facultad de Medicina Veterinaria Y Zootecnia, Centro de Enseñanza, Investigación Y Extensión en Ganadería Tropical, Universidad Nacional Autónoma de México, Veracruz, Mexico; 5 Facultad de Medicina Veterinaria y Zootecnia, Universidad Autónoma de Sinaloa, Culiacán, México

**Keywords:** Methane, Canola oil, Cattle, Grazing, Digestion

## Abstract

Four rumen-cannulated cows (*Bos taurus* × *Bos indicus*, 657 ± 92 kg body weight, BW) in a rotational grazing (*Urochloa* sp.) system were assigned to different canola oil (CO) inclusion levels, 0.0, 0.40, 0.80, and 1.2 g/kg according to shrunk body weight (SBW, BW adjusted for gastrointestinal filling) in a 4 × 4 Latin Square design to evaluate CO on the CH_4_ emissions and dietary energy intake. CH_4_ emissions were estimated using an infrared analyzer methodology (*Sniffer* method). Grass intake and fecal production were estimated using Cr_2_O_3_ as an external marker. CO supplementation increased (linear effect, *P* ≤ 0.05) total dry matter and gross energy intake with a linear increase (*P* = 0.09) in neutral detergent fiber (NDF) intake. While digestible energy (Mcal/kg) linearly increased with increasing CO supplementation level (linear effect, *P* < 0.05), total tract digestion of organic matter, NDF, and CP was comparable (*P* > 0.05) between levels. Maximal CO supplementation (1.2 g/kg SBW) significantly decreased total ruminal protozoa population, acetate:propionate ratio, and enteric methane production (g/kg DMI) by 9, 5.3, and 17.5%, respectively. This study showed that, for cows grazing tropical forages, CO can be supplemented up to 1.2 g/kg SBW (5.8% of the total diet) without negatively affecting intake and nutrient digestion while reducing ruminal fermentation efficiency and enteric methane emission (≤ 17.5%).

## Introduction

Livestock is among the highest contributors to greenhouse gas (GHG) emissions, mainly methane (CH_4_) globally, with extensive grazing production systems having the highest emissions (Hristov et al., 2013a; Herrero et al., 2016).

The CH_4_ emission represents a significant loss of energy intake (Audsley and Wilkinson, 2014; Hristov et al., 2015). Therefore, the challenge is to develop diets and strategies to reduce CH_4_ emission by optimizing energy use in ruminant diets, consequently improving productive performance and reducing their environmental impact (i.e., global warming; McGeough et al., 2010; Wu et al., 2016).

Previous reports have pointed out to the addition of lipids to reduce CH_4_ emissions in ruminants (Grainger and Beauchemin, 2011; Hristov et al., 2013b; Knapp et al., 2014; Martin et al., 2016). Unsaturated fatty acids (UFA) decrease CH_4_ emission by increasing propionic acid production and decreasing protozoa population and UFA hydrogenation (Bonilla and Lemus, 2012, Mata e Silva et al., 2017). These effects have been observed using long-chain (C18) UFAs (i.e., vegetable and fish oils; oleic (C18:1) and linoleic (C18:2)) and medium chain fatty acids (Patra, 2013; Yanza et al., 2021). Studies using CO supplementation in grazing cattle (46 g/kg CO sprayed onto *Lolium perenne* meadow grass; Piñares-Patiño et al., 2016) and a barley silage diet (75% of DMI with 4.6% CO; equivalent to 1.2 g oil/kg SBW; Beauchemin and McGinn, 2006) resulted in a decrease of 11% and 32% CH_4_ emissions, respectively. Moreover, in grazing cattle, there was a decrease in ruminal acetate:propionate ratio (Piñares-Patiño et al., 2016). It was suggested that the reduction in CH_4_ may be due to a decrease in feed intake and total DM digestibility of the tract as a direct consequence of the decrease in dNDF. The effects of oil supplementation have been reported primarily under temperate climate conditions with scarce reports for cattle under tropical grazing conditions.

Tropical pastures have low dNDF, nitrogen (N), and energy concentration which have a detrimental effect on N utilization efficiency and carbon retention, causing an increase in the production of enteric CH_4_ in cattle grazed in tropical grazing systems. To reduce enteric CH_4_ emissions significantly in these conditions, a suitable GHG mitigation strategy is the use of concentrates supplemented with adequate amounts of vegetable oils to increase energy intake but with a minimal impact on forage dNDF. Mata e Silva et al. (2017) reported a 23% reduction in CH_4_ emissions in dairy cows (Holstein × Gyr) grazing *Urochloa* sp. and supplemented with 2.86 kg/day concentrate and 13.4% sunflower oil (0.73 g of oil/kg of body weight). However, these authors did not consider ruminal fermentation variables in their study. It is important to point out that oil supplementation at high levels in grazing cattle should be used carefully due to possible detrimental effects on ruminal digestible NDF (dNDF; Jenkins and Palmquist, 1984), low intestinal digestibility, and energy value of lipids (Zinn and Jorquera, 2007).

In beef cattle, optimal FA digestibility is limited to a maximum lipid intake of 0.96 g/kg body weight (BW), representing an adequate value of energy in fat (Zinn and Jorquera, 2007). However, other studies have observed similar effects on CH_4_ at lower oils supplementation levels (~ 0.73 g/kg BW; Mata e Silva et al., 2017). Consequently, it is necessary to determine the optimal level of vegetable oil inclusion to reduce CH_4_ emissions without decreasing dry matter intake (DMI) and dietary energy use in grazing cattle under tropical conditions. In this context, this study aimed to evaluate the effect of the inclusion level of CO on nutrient digestibility, ruminal fermentation, and enteric CH_4_ emissions in grazing cattle under tropical conditions supplemented with a fixed amount of commercial concentrate.

## Materials and methods

### Location

The study was carried out at the Center for Teaching, Research and Extension in Tropical Livestock (CEIEGT, FMVZ-UNAM), located in Tlapacoyan, Veracruz, Mexico (20°03′N and 93°03′W). The climate is warm humid, Af (m) w"(e) (García, 2004), with an average daily temperature of 23.9 ± 0.5 °C and an average annual rainfall of 1931 ± 334 mm.

### Animals, feeding, and experimental design

Four non-pregnant, non-lactating rumen-cannulated adult crossbred cows (*Bos taurus* × *Bos indicus*) with an average shrunk body weight (SBW; BW adjusted for gastrointestinal filling) of 631 ± 88 kg were used in the study. At the start of the experiment, cows were weighed for three consecutive days at 1100 h. The SBW was obtained by multiplying BW by a correction factor of 0.96 to account for intestinal filling, according to the National Academies of Sciences, Engineering and Medicine (NASEM, 2016), and used previous studies (Cannas et al., 2004; Estrada-Angulo et al., 2018; Rivera-Villegas et al., 2019).

Cows were housed in 1 hectare of a *Urochloa* sp. grazing paddock, divided into four pasture sections of 50 × 50 m. Feeding was based on a rotational grazing system of 7 days per paddock, with a recovery period of 21 days, ensuring an average height of the pasture of 40 cm before grazing and 10 cm of residual. Animals received 7 kg/cow (as-fed basis) of commercial concentrate (Purina®, Puricarne sequia) daily supplemented with CO (Cristal® 99% canola oil, Aceites, Grasas y Derivados, S.A., Jalisco, Mexico) at four different levels: 0.0, 0.40, 0.80, and 1.20 g CO/kg SBW, resulting in a final concentration of 0, 40, 80, and 120 g CO/kg of concentrate (on a DM basis). The concentrate was offered in equal amounts (3.50 kg as-fed basis) at 0600 and 1500 h. The residual concentrate was introduced directly into the rumen through the ruminal cannula to ensure expected CO inclusion. The average DM content in CO-supplemented concentrate was 89.07 ± 0.7%, with an average DM intake of 6.235 kg/cow, so the total daily intake of CO was 0, 249, 499, and 748 g per animal, corresponding to 0.0, 0.40, 0.80, and 1.20 g CO/kg SBW, respectively (Zinn and Jorquera 2007; Bayat et al., 2018).

The experiment design was a Latin Square, with four periods for four treatments; where each period lasted 19 days (13 for adaptation and 6 for sampling feces and ruminal content).

### Sampling

#### Feed

Forage samples were hand-plucked (Cook, 1964) every 7 days; when the animals changed paddock, the samples were weighed fresh and placed in a forced air oven at 65 °C until constant weight; once dry, the samples were stored until analysis in the laboratory. Forage sampling was carried out by collection samples from > 1 paddock followed by mixing and selection of a representative sample according to the percentage of time spent on these paddocks for analysis. Concentrate and forage samples were dried (60 °C for 72 h), ground, and sieved (1 mm) for further composition analysis. CO samples were stored protected from light (amber bottles) and under cool conditions (18 °C) until analysis.

To determine forage intake (and nutrient digestibility), cows received 10 g of Cr_2_O_3_ (68.52% purity) contained in a cellulose paper envelope and placed daily directly into the rumen through the cannula from day 8 to 17 of each period at the same time (1530 h). Individual fecal samples (200 g) were collected directly from the rectum every 8 h (3 times daily) during the last 4 days of each treatment period, as follows: d1 00:00, 08:00, and 16:00; d2 02:00, 10:00, and 18:00; d3 04:00, 12:00, and 20:00; d4 06:00, 14:00, and 22:00 h. Fecal samples were refrigerated (1–4 °C) and homogenized at the end of each sampling period to generate a 400 g composite sample which was then frozen (− 18 °C) for further chemical analysis.

#### Ruminal liquor

Samples of ruminal liquor (200 mL) were collected on days 18 and 19 of each experimental period every 4 h. These were filtered through eight gauze layers and the pH measured using a portable potentiometer (Oakton, pHTestrs®).

For protozoa number estimation, a 5 mL of filtered ruminal fluid vial was collected and 5 mL of iodine solution added (1.5 g of potassium iodine and 0.5 g of re-sublimated iodine brought to 100 mL). Samples were stored at 1–4 °C before protozoa estimation was carried under a binocular microscope (Carl Zeiss Axiolab E re, Artisan Technology Group, Champaign, IL, USA) using a hemocytometer (Neubauer improved, Marienfeld, Germany). Six replicates of the same sample were made to estimate the number of protozoa per milliliter.

Forty milliliters of ruminal liquor (corresponding to 4-h post-feeding, i.e., 1900 h concentrate, and 0300 h forage) was collected in containers with 10 mL 25% (v/v) metaphosphoric acid and frozen (− 18 °C) for ammonia–nitrogen and VFA profile determination. Ammonia–nitrogen (NH_3_-N) was estimated by micro diffusion as described by Conway and O’Malley (1942) and VFA profile was measured by gas chromatography (Perkin Elmer, AutoSystem XL model).

#### Methane emissions estimation

CH_4_ was measured using the method described by Garnsworthy et al. (2012). Briefly, CH_4_ concentrations were measured when the concentrate was offered at 0600 and 1500 h using an infrared CH_4_ analyzer (0–10 ppm; Guardian Plus; Edinburgh Instruments Ltd., Livingston, UK). The sampling tube (6 mm internal diameter) was placed in the middle of the closed trough (at the height of the nose) in the form of a ring to prevent blockages. CH_4_ concentration was recorded every second on a data logger (Simex SRD-99; Simex Sp. z o.o., Gdansk, Poland) and then visualized using logging software (Loggy Soft; Simex Sp. z o.o.). Feeder entry times and cow identification were recorded manually. Raw data from the logger were transformed into values ​​for peak height (maximum minus baseline CH_4_ concentration for each eructation) and area under the curve (AUC) for each peak (representing total CH_4_ release per eructation). Peaks were identified and quantified and peaks’ values less than 200 ppm below baseline were discarded. For each feeder entry, mean peak height and AUC were calculated, along with peak frequency (belching rate). A CH_4_ emission rate (MER) during concentrate feeding (i.e., feeder entry) was calculated as the product of the maximum frequency and the maximum mean area. The CH_4_ emission estimations were calculated as the average of 12 sampling events, i.e., 6 sampling days with two daily samples at 0600 and 1500 h. Methane emission was expressed as total production, g/day; CH_4_ yield, g/kg DMI and g/kg of digested NFD. The methane conversion factor (extent to which feed energy is converted to CH_4_, Ym) was calculated according to IPCC Tier 2 (2006); the energy conversion from methane to flux in mass units was 55.56 MJ/kg (Lassey, 2007). As follows:

$${\mathrm{Y}}_{\mathrm{m}}=\frac{\frac{\left({\sum }_{i=}^{n}{\mathrm{CH}}_{4i}\right)}{\mathrm{n}}}{\frac{\left({\sum }_{i=1}^{n}{\mathrm{GEI}}_{i}\right)}{n}}\times 100$$

where Ym, methane conversion factor (% of GE Intake); CH4*i*, the *i*th observed methane energy emissions (MJ/day); GEI*i*, the *i*th observed Gross Energy Intake (MJ/day); and *n*, number of observations (Kaewpila and Sommart, 2016). The means used for the calculation are those obtained in the experiment. Mcal was multiplied by 4182 to get MJ.

### Sample analysis

Feed ingredients (CO, *Urochloa* sp., and concentrate) and faces samples were analyzed for dry matter content (DM, method 934.01; AOAC, 2015), crude protein (CP, method 2001.11; AOAC, 2015), ash (method 942.05; AOAC, 2015), neutral detergent fiber (NDF), and acid detergent fiber (ADF), as described by Van Soest et al. (1991), using alpha-amylase without correction for ash and an ANKOM 2000 analyzer, Gross Energy using the calorimetric pump approach (Parr, 6400; IL, USA).

Forage DMI was estimated (Schneider and Flatt, 1975) considering Cr_2_O_3_ intake/Cr_2_O_3_ fecal concentration. The digestibility of *Brachiaria* sp. and concentrate samples was determined by a previously validated in vitro procedure (Goering & Van Soest, 1970). To obtain fecal output from forage, fecal production associated with concentrate and CO was discounted from the total fecal output with the Cr_2_O_3_ (Oliveira et al., 2007). Cr_2_O_3_ in fecal samples was determined using atomic absorption spectrometry following Williams et al. (1962) methodology.

### Statistical analysis

Data were analyzed as a 4 × 4 Latin Square Design using the proc MIXED procedure of SAS 9.0 (Statistical Analysis System, Inc., Cary, NC, USA), according to the following model: *Y*_*ij*_ = *μ* + *H*_*i*_ + *C*_*j*_ + *Τ*_*k*_ + *ε*_*ij*_, where *Y*_*ijk*_ is the dependent variable, *μ* is the general mean, *H*_*i*_ is the effect of the *i*th animal (row), *C*_*j*_ is the effect of the *j*th period (column), *T*_*k*_ is the effect of the *k*th CO supplementation, and *ε*_*ijk*_ is the residual effect. Linear and quadratic trends in response to the level of inclusion of CO by using orthogonal polynomial contrasts (Cochran and Cox, 1992).

For ruminal fermentation parameters (VFAs, protozoa number estimation, and CH_4_ emission), sampling period/time was included as a repeated measure; differences with *P* values ≤ 0.05 were considered statistically significant. Normality of distribution and homogeneity of variance for residuals were tested using proc univariate procedure of SAS 9.0.

## Results

Results for the chemical composition analysis of CO, commercial concentrate, and *Urochloa* sp. grass are shown in Table 1. Supplemental CO represented 0.0, 1.97, 4.15, and 5.81% inclusion of the total DM intake (CO supplemented concentrate + forage) for 0.0, 0.40, 0.80, and 1.20 g/kg SBW treatments, with a total fat concentration of 2.66, 4.53, 6.67, and 8.30%, respectively.

**Table 1:** Chemical compositions of the ingredients fed to crossbred (*Bos taurus* × *Bos indicus*) cows

Items (g/100 g of DM)	Canola oil	*Urochloa* sp.	Commercial concentrate
Crude protein	-	6.4	19.5
Ashes	-	11.1	13.7
Neutral detergent fiber	-	66.6	41.8
Acid detergent fiber	-	41.4	14.6
Crude fat	99.0	2.26	3.0
Gross energy (Mcal/kg of DM)	9.5	3.7	3.7

*DM*, dry matter

### Dry matter intake and digestion

The effect of CO on feed intake, fecal excretion, and total tract digestion is shown in Table 2. The average DMI of concentrate was 5870 g/cow/day, while CO intake was 0.00, 249, 499, and 748 g. Daily forage DMI (grazing *Urochloa* sp.) was not affected by treatments averaging 6030 g. As expected, intake of the CO-supplemented concentrate was comparable between treatments, and intake of CO increased linearly (*P* < 0.01) according to supplementation level. It was noted that total DMI increased as the CO supplementation increased in a linear fashion (*P* = 0.02). There were no treatment effects on organic matter (OM) or CP intake, but NDF intake tended (*P* = 0.09) to increase as CO was increased in the diet. There were no treatment effects on total tract digestion of organic matter, NDF, or CP, but digestible energy (Mcal/kg) was linearly increased with CO supplementation.

**Table 2:** Dry matter intake, fecal excretion, and total tract digestion of cows fed with different levels of canola oil

Items	Canola oil g/kg SBW^1^				SEM^2^	*P*-value^3^	
	0 .00	0.40	0.80	1.20		L	Q
*Intake*							
Concentrate, kg/day	6.23	5.99	5.74	5.49	0.10	0.02	0.77
Canola oil, kg/day	0	0.25	0.50	0.75	0.16	< 0.01	1.00
Concentrate plus canola oil, kg/day	6.23	6.24	6.24	6.23	0.12	0.56	0.87
*Urochloa* sp., kg/day	5.34	6.37	5.78	6.62	0.29	0.11	0.81
Total DMI, kg/day	11.6	12.6	12.0	12.9	0.42	0.02	0.81
OM, kg/day	10.3	11.2	10.7	11.4	0.25	0.11	0.76
NDF, kg/day	6.47	7.13	6.81	7.45	0.21	0.09	0.97
ADF, kg/day	3.26	3.68	3.44	3.85	0.13	0.11	0.98
CP, kg/day	1.56	1.58	1.49	1.54	0.02	0.75	0.22
GE, Mcal/day	45.0	52.0	52.0	57.0	2.51	< 0.01	0.78
*Fecal excretion*							
DM, kg/day	4.19	4.39	4.20	4.36	0.16	0.02	0.81
OM, kg/day	3.39	3.44	3.41	3.50	0.04	< 0.01	0.13
NDF, kg/day	2.27	2.57	3.03	3.85	0.10	0.08	0.34
ADF, kg/day	1.94	2.08	1.86	2.30	0.06	0.22	0.41
CP, kg/day	0.46	0.49	0.45	0.49	0.01	0.17	0.82
GE, Mcal/day	16.4	18.9	19.3	21.1	0.84	< 0.01	0.78
*Total tract digestion*							
DM, %	63.8	65.2	65.1	66.1	0.47	0.22	0.51
OM, %	66.9	69.3	68.0	69.2	0.57	0.78	0.66
NDF, %	64.6	64.0	55.5	48.3	4.02	0.06	0.58
ADF, %	40.6	44.3	45.9	40.3	1.25	0.97	0.28
CP, %	70.3	68.6	69.5	68.5	0.42	0.34	0.74
DE, %	63.5	63.7	62.8	63.1	0.21	0.67	0.98
DE, Mcal/day	28.8	32.5	32.9	36.3	1.53	< 0.01	0.85
DE, Mcal/kg	2.47	2.62	2.71	2.82	0.04	< 0.01	0.75

^1^*SBW*, shrunk body weight, BW × 0.96 (NASEM, 2016); ^2^*SEM*, standard error means; ^3^*P*-value L (linear) and Q (quadratic); *DMI*, dry matter intake; *DM*, dry matter; *OM*, organic matter; *NDF*, neutral detergent fiber; *ADF*, acid detergent fiber; *CP*, nitrogen; *GE*, gross energy; *DE*, digestible energy; *Mcal*, megacalories; intake, was estimated based on fecal output (using Cr_2_O_3_ as marker)

### Ruminal fermentation

The effects of CO supplementation on NH_3_-N, pH, protozoa number, and VFA are shown in Table 3. Different CO supplementation levels showed similar levels (*P* > 0.05) of ruminal N-NH_3_ (25.53 and 34.37 mg/dL) and ruminal pH values (6.47 and 6.50). Total ruminal protozoa population decreased as CO supplementation increased: 29.7, 69.5, and 88.6% (linear effect *P* < 0.01) for 0.4, 0.8, and 1.2 g CO/kg SBW, respectively. This decrease was most evident in the *Entodinidae* (31.4, 68.8, and 88.8%, respectively) and *Holotrichidae* (11.2, 77.5, and 86.4%) populations.

**Table 3:** Effect of the inclusion of canola oil on rumen fluid ammoniacal nitrogen, pH, protozoa, volatile fatty acids, and methane

Items	Canola oil g/kg SBW^1^				SEM^2^	*P*-value^3^	
	0 .00	0.40	0.80	1.20		L	Q
NH_3_-N, *mg/dL*	25.5	26.7	33.0	34.4	2.23	0.27	0.99
pH	6.49	6.50	6.47	6.48	0.01	0.78	0.99
*Protozoa,* × *10*^*4*^* cells/mL*							
*Entodinidae*	1845	1265	575	207	364	< 0.01	0.05
*Holotrichidae*	167	148	37	23	37	< 0.01	0.79
Total protozoa	2012	1413	612	230	401	< 0.01	0.05
*Volatile fatty acids*							
TVFA, m*M*	77.5	74.5	74.3	70.7	1.39	0.18	0.92
Acetic, mol/100 mol	60.1	58.4	57.6	55.5	0.96	< 0.01	0.73
Propionic, mol/100 mol	19.1	20.9	22.6	23.8	1.30	< 0.01	0.50
Butyric, mol/100 mol	11.8	11.7	10.8	11.1	0.24	0.76	0.36
Valeric, mol/100 mol	3.32	3.33	3.44	3.42	0.03	< 0.01	0.38
Iso-butyric, mol/100 mol	2.60	2.58	2.51	2.68	0.04	0.90	0.41
Iso-valeric, mol/100 mol	2.10	2.07	2.01	2.14	0.03	0.06	0.79
A:P^4^	3.14	2.79	2.54	2.33	0.17	< 0.01	0.47
*Infrared methane emissions*							
MER, mg/min	0.92	0.85	0.82	0.49	0.10	0.01	0.25
CH_4_, g/day	304.8	300.9	299.1	280.1	5.51	0.01	0.25
CH_4_, g/kg DMI	26.3	23.9	24.9	21.7	0.98	0.01	0.65
CH_4_, g/kg DNDF	72.6	66.0	79.1	77.8	1.99	0.74	0.44
CH_4_, % GEI (Ym)	9.03	7.94	7.86	6.70	0.48	0.01	0.88

^1^*SBW*, shrunk body weight, BW × 0.96 (NASEM, 2016); ^2^*SEM*, standard error means; ^3^*P*-value linear and quadratic; *NH*_*3*_*-N*, ammonia–nitrogen; *TVFA*, total volatile fatty acids; ^4^*A:P*, acetic acid to propionic acid ratio; *MER*, methane emission rate; *CH*_*4*_, methane; *DMI*, dry matter intake; *DNDF*, digested neutral detergent fiber; *GEI*, gross energy intake. Ym = CH_4_ energy loss, in proportion to the GEI

No differences were observed in total ruminal concentrations of VFA as well as butyric, iso-butyric, and iso-valeric acids. Furthermore, acetic acid decreased linearly (*P* < 0.01) to 7.7%, whereas propionic and valeric acids increased (*P* < 0.01) to 24.6 and 3.0%, respectively, with CO supplementation compared to the control. Consequently, the acetate:propionate ratio decreased by 10.7, 18.6, and 25.2% for the levels of 0.40, 0.80, and 1.20 g CO/kg SBW, respectively.

### Methane emissions

The enteric emissions of CH_4_ are shown in Table 3. Compared with the diet without canola oil supplementation (0.0 g/kg SBW), CH_4_ emission rate (MER) decreased linearly (7.6, 10.9, and 46.7%, *P* < 0.05) as the CO level in the diet increased. For CH_4_ expressed in g/day, the observed decrease (linear effect, *P* < 0.05) was 1.28, 1.87, and 8.1%, whereas for CH_4_ described as g/kg DMI, the reduction (linear effect, *P* < 0.01) was 9.1, 5.3, and 17.5%. Moreover, CH_4_ production per kilogram of digested NDF was similar (*P* > 0.05) between treatments. For the methane conversion factor Ym (CH4, % GEI), a linear decrease (*P* < 0.01) of 12.1, 13, and 25.8% was observed as the CO level increased.

## Discussion

This study demonstrated that increasing levels of CO supplementation to the diet of grazing beef cows did not affect digestibility or ruminal fermentations parameters but were able to decrease CH_4_ emission by up to 17.5% in animals receiving the highest CO supplementation (1.2 g CO/kg SBW). Moreover, we observed that these effects had a linear effect with no impact on DMI in animals receiving or not CO supplementation.

### Chemical composition of *Urochloa* sp

The chemical composition of *Urochloa* sp. agrees with that reported in previous studies considering a similar state of maturity (Avellaneda Cevallos et al., 2008). The nutrient concentration in the commercial concentrate closely matched that specified on the label provided by the feed manufacturer.

### Intake and digestibility

Animals in this study had a fat intake of 2.66, 4.53, 6.67, and 8.30% corresponding to the CO supplementation of 0.0, 0.40, 0.80, and 1.20 g CO/kg SBW respectively. Although animals at the highest CO supplementation level had a fat intake of 8.3%, we did not observe any effect on intake. It has been indicated that lipid supplementation above 7% DM generally decrease intake due to an increase in circulating UFA that cause receptor activation in the satiety center of the hypothalamus, and a potential reduction in fiber digestibility delaying passage rate (Allen, 2000; Kumar et al., 2014). Several mechanisms can explain how dietary lipids interfere with ruminal fermentation, such as when long-chain FA (present in CO), escape biohydrogenation and are potentially toxic to bacteria responsible for fiber degradation (cellulolytic, gram +) and for methanogens and protozoa (Desbois and Smith, 2010; Zeitz et al., 2013). Furthermore, fats form a physical layer on the fiber, preventing microbial degradation, which has a detrimental effect on voluntary intake (Jenkins, 1993). However, restriction of supplemental fat levels based on percentage of inclusion in the diet of ruminants is a limited concept. Currently, a better indicator used is the total lipid intake/kg of live weight with the recommended amount of 1 g of lipid intake/kg of weight (Zinn and Jorquera, 2007). In our experiment, the proportion of lipid intakes was 0.49, 0.91, 1.27, and 1.68 g/kg BW for supplementation levels of 0.0, 0.40, 0.80, and 1.20 g CO/kg SBW, respectively. Therefore, it was expected a potential reduction in DMI with 0.80 and 1.20 CO/kg SBW treatments.

Nonetheless, the effects of adding supplemental oils on the intake and digestibility of fresh forage-based diets have been inconsistent. Some studies on oil supplementation of forage-based diets indicate reductions in both feed intake and overall diet digestibility (Beauchemin and McGinn 2006; Pavan et al., 2007; Cosgrove et al., 2008), while other studies found no effect on intake (King et al., 1990; Woodward, 2006). In general, concentrate offer to grazing animals reduces grass intake due to a substitution effect but the total DMI increases (Vazquez and Smith, 2000). Increasing the CO supplemental level increased the DMI up to 16% at the highest level of supplementation (Table 2), increasing GE (Mcal/day) intake by up to 26%. The effect of increased DMI, observed in the present study, agrees with Piñares-Patiño et al. (2016) in steers grazing temperate pastures, who reported an increase in DMI of 9.4%. In an early report, Reid (1959) indicates that the constant addition of supplemental oil to grazed grass may not produce abrupt changes in the rumen environment and function; thus, DM intake must not be negatively affected.

The main factor that limits weight gain in adult ruminants in grazing is energy. Inclusion of oils in the diet increases energy density and total tract energy digestible because fats are highly digestible and contain more than twofold more energy than concentrates. Nutrient digestibility was not affected by the addition of CO, except for an increase in energy (Mcal/kg; *P* < 0.05) as the addition of CO increased. Ueda et al. (2003) reported that compared to un-supplemented cows, ruminal OM and fiber digestibility increased markedly with 3% LSO supplementation (equivalent to 1.55 oil/kg BW) in cows on a forage-rich diet, with no effect on total tract digestibility of OM and NDF. Thus, at equal intakes, cattle that receive supplemental oils have greater energy availability even when the digestibility of some fractions (i.e., NDF) may be affected.

Although the main objective of this experiment was to determine the role of supplemental oil on the energy intake and decrease of methane production in cattle under tropical conditions, oil supplementation in grazing cattle under tropical conditions may be cost prohibitive. However, tropical plants such as *Moringa oleifera* seed, which has up to 40% oil (Leone et al., 2016), may be an alternative for sustainable livestock production systems that allow increasing the energy intake of the diet while reducing methane emissions.

### Ruminal fermentation

The values of rumen parameters are in agreement with those reported by Ribiero et al. (2016), in Nellore cattle with a diet based on sugarcane with different portions of concentrate (30 to 80) for N-NH_3_ (18.7 to 30 mg/dL), pH (6.49 to 6.01), and total protozoa (2127 to 1748 10^4^/mL). The addition of FA can have a toxic effect on rumen protozoa (Machmüller and Kreuzer, 1999); according to Onetti et al. (2001), the numbers of protozoa in rumen decreased when fat was added. In the present study, the ratio of acetate:propionate decreased significantly with the addition of canola oil, which is consistent with the findings of Beauchemin and McGinn (2006) where despite this decrease in the acetate ratio and the increase in propionate, there were no found effects in the decrease of methane.

### Methane emissions

Variations in the values of methane emissions among studies are mainly due to the technique used to measure methane and the chemical composition of the forage consumed. Enteric methane emission (g CH_4_/kg DMI) in the control non-CO supplemented cows in this study was 26.3 g CH_4_/kg DM (range 21.7–26.3 g CH_4_/kg DMI), which is close to the values determined by Kennedy and Charmley (2012) for cattle consuming pastures and tropical legumes (19.6 g/kg DMI) and by Beauchemin and McGinn (2006; 20 g CH_4_/kg DMI) for cattle on a barley silage (75%) and grain diet (steam-rolled barley, 19%). However, slightly lower than the 28 g of CH_4_/kg DMI reported for cattle grazing *Leucaena* (McGinn et al., 2011). It is evident that the methodology employed in this study would produce results similar to those previously reported for cattle under comparable conditions.

Estimated methane emissions in grazing cows in this study reduced linearly according to CO inclusion level in the diet, with the highest inclusion reducing nearly 17% daily methane output. Several studies have reported that lipids reduce CH_4_ production in the rumen by different mechanisms (Dohme et al., 2001; Machmüller et al., 2001; Bayat et al., 2018; Muñoz et al., 2019; Nogueira et al., 2020). Similar to our findings, Moate et al. (2011) showed that lactating cows’ diets (60:40 ratio of forage: concentrate) containing 51, 52, and 65 g fat/kg DM decreased emissions of CH_4_ (g/day) in a linear fashion (7.6, 4.5 and 10.2%, respectively), compared to a control diet. Beauchemin et al. (2007) reported that the addition of sunflower oil, rich in linoleic acid (C18:2), was effective in reducing the emissions of CH_4_ by 14% (177.4 vs. 152.7 g/day) and 11.5% (20 vs. 17.7 g/kg DMI) when added to 3.4% inclusion. A study by Woodward (2006), carried out with lactating dairy cows fed pasture forage dominated by perennial ryegrass (13.5–15.0 kg DMI/day) supplemented with 500 g of fish oil, reported a reduction (compared to the control) in the CH_4_ yield of 27%. Overall, these studies show that addition of fatty acids (fats or oils) to cattle diets seems to have decreasing effect on CH_4_ emissions regardless of the source and the feed that animals are offered.

In our study, the rate at which CH_4_ decreased was 3.6% per each 1% oil added to the diet. Consistent with this, Martin et al. (2010) in a metanalysis with 67 in vivo diets for sheep, beef, and dairy cattle estimated an average 3.8% decrease in enteric CH_4_ (g/kg DMI) with each of 1% added fat. From a similar meta-analysis study on cattle, for each percentage increase of fats, methane emissions decline by 13.4 g/day (i.e., 3.77% compared with diets without fat) or 0.66 g/kg DMI (*n* = 105) within dietary fat concentrations of 1.24–11.4% (Patra, 2013). In a recent meta-analysis study in dairy cattle (12 studies including 362 individual measurements) under an standard TMR diet containing 30 g of lipids/kg DMI, researchers estimated that for each 1% addition of fat in the diet, methane emissions decreased by approximately 3.5% (Moate et al., 2016). Hristov et al. (2013a) consider that concentrates can influence the reduction of enteric CH_4_ from 35 to 40% of inclusion, but at higher inclusion levels (> 40%), a decrease in fiber digestibility may be observed. In this trial, the decrease in methane could partially be explained by a direct effect of oil on ruminal methanogenesis, and although there was a reduction of digestibility of ruminal NDF, this considering that oil supplementation tended (*P* = 0.06) to reduce total tract NDF digestion and greater than 90% of total NDF digestion occurs in the rumen (Huhtanen et al., 2010); in this trial, the decrease in methane was not due to the amount of NFD digested (Table 3).

The decrease in methane emission is also evaluated as CH_4_ energy loss, in proportion to the gross energy intake (GEI), a variable known as Ym, which is important to determine the effect on the efficiency of energy utilization of the animal (Beauchemin et al. 2022). Enteric methane emission represents a loss of 2 to 12% of GEI (Johnson and Johnson, 1995). The IPCC (2006) (tier 2 level) use the conversion factor Ym = 6.5% + 1% of GEI for nonfeedlot cattle (fed concentrate diet < 90% of total intake). However, it was developed from a dataset on *Bos taurus* fed temperate. Using various studies to estimate models in tropical conditions, the average Ym value was 5.84% ranging from 1.96 to 10.6 (Patra, 2017). Kaewpila and Sommart (2016) indicate that the value in tropical conditions is 8.4% ± 0.4%. Tee et al. (2022) estimated the Ym value of 8.3%, in tropical beef cattle systems and when fed diets with higher forage content. In our study, we observed a range between 6.7 and 9.03% (average 7.9%), and it decreased to 6.7% with the highest inclusion of CO. It will be important to develop models that estimate the methane conversion factor, considering different dietary situations such as dietary lipid concentration.

We concluded that canola oil supplemented at 1.2 g CO/kg SBW (5.4%) in cows grazing tropical fodder and supplemented with a fixed amount of commercial concentrate increases the total energy intake and efficiency of rumen fermentation, thereby increase the DE value (Mcal/kg) of the diet by up to 12.4% and reducing the emission of enteric methane by up to 17.5%. Therefore, alternative sources of oil, such as tree seeds, should be sought in sustainable production systems under tropical conditions.

## Data Availability

The data supporting this study’s findings are available from the corresponding author upon reasonable request.
